# Genetic structuring and migration patterns of Atlantic bigeye tuna, *Thunnus obesus *(Lowe, 1839)

**DOI:** 10.1186/1471-2148-8-252

**Published:** 2008-09-17

**Authors:** Elena G Gonzalez, Peter Beerli, Rafael Zardoya

**Affiliations:** 1 Department of Biodiversity and Evolutionary Biology, Museo Nacional de Ciencias Naturales, CSIC, José Gutiérrez Abascal, 2; 28006 Madrid, Spain; 2 Department of Biochemistry and Molecular Biology IV, Universidad Complutense de Madrid, 28040, Madrid, Spain; 3 Department of Scientific Computing, Florida State University, Tallahassee, FL, 32306-4120

## Abstract

**Background:**

Large pelagic fishes are generally thought to have little population genetic structuring based on their cosmopolitan distribution, large population sizes and high dispersal capacities. However, gene flow can be influenced by ecological (e.g. homing behaviour) and physical (e.g. present-day ocean currents, past changes in sea temperature and levels) factors. In this regard, Atlantic bigeye tuna shows an interesting genetic structuring pattern with two highly divergent mitochondrial clades (Clades I and II), which are assumed to have been originated during the last Pleistocene glacial maxima. We assess genetic structure patterns of Atlantic bigeye tuna at the nuclear level, and compare them with mitochondrial evidence.

**Results:**

We examined allele size variation of nine microsatellite loci in 380 individuals from the Gulf of Guinea, Canary, Azores, Canada, Indian Ocean, and Pacific Ocean. To investigate temporal stability of genetic structure, three Atlantic Ocean sites were re-sampled a second year. Hierarchical AMOVA tests, *R*_*ST *_pairwise comparisons, isolation by distance (Mantel) tests, Bayesian clustering analyses, and coalescence-based migration rate inferences supported unrestricted gene flow within the Atlantic Ocean at the nuclear level, and therefore interbreeding between individuals belonging to both mitochondrial clades. Moreover, departures from HWE in several loci were inferred for the samples of Guinea, and attributed to a Wahlund effect supporting the role of this region as a spawning and nursery area. Our microsatellite data supported a single worldwide panmictic unit for bigeye tunas. Despite the strong Agulhas Current, immigration rates seem to be higher from the Atlantic Ocean into the Indo-Pacific Ocean, but the actual number of individuals moving per generation is relatively low compared to the large population sizes inhabiting each ocean basin.

**Conclusion:**

Lack of congruence between mt and nuclear evidences, which is also found in other species, most likely reflects past events of isolation and secondary contact. Given the inferred relatively low number of immigrants per generation around the Cape of Good Hope, the proportions of the mitochondrial clades in the different oceans may keep stable, and it seems plausible that the presence of individuals belonging to the mt Clade I in the Atlantic Ocean may be due to extensive migrations that predated the last glaciation.

## Background

Marine pelagic fishes show broad geographic distribution, large population sizes, and highly migratory movements that are thought to ultimately result in little genetic structuring (e.g. [1-4]). The above-mentioned biological peculiarities result in complex phylogeographic patterns (e.g. [5,6]), which do not often meet the assumptions (e.g. uniform effective population sizes or symmetric effective migration rates) of classic statistics that measure gene flow (*F*_*ST*_) [7-9], and thus make the study of population genetic variation of marine pelagic fishes particularly challenging. In addition, philopatric behavior, local larval retention mechanism, and historical processes such as e.g. the effect of Pleistocene sea level changes also complicate estimation of genetic differentiation for many marine pelagic fishes [2,10,11].

Given this complexity, accurate determination of marine pelagic fish genetic structuring would ideally require both temporal (different years) and spatial (covering large areas) sampling, analysis of multiple loci (including both mitochondrial, mt, and nuclear data) that improves the estimates considerably [12,13], as well as complementation of classic summary statistics with more recent coalescent-based approaches [14] that convey statistical efficiency and flexibility to the estimation of population genetic parameters by using maximum likelihood (ML) [15-18] and Bayesian inference (BI) [19-21].

**Figure 1:**
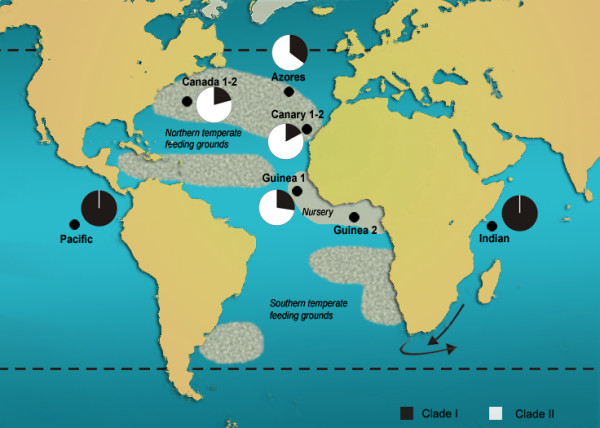
**Locations of bigeye tuna samples in the Atlantic Ocean, Indian Ocean and Pacific Ocean (solid circles).** The textured grey areas show potential feeding grounds at the North and the South, respectively. The arrows indicates the Agulhas Current and its retroflection at the Cape of Good Hope (modified from [29]). Dashed dark lines show the limits of bigeye tuna geographic distribution. The relative proportions of mitochondrial control region Clade I (black) and Clade II (white) for each population of *T. obesus *at the Atlantic Ocean, the Indian Ocean and the Pacific Ocean [10] are also indicated.

Bigeye tuna, *Thunnus obesus *(Lowe, 1839) is a marine pelagic fish species characterized by large populations and a worldwide distribution (Atlantic Ocean, Indian Ocean and Pacific Ocean) restricted to tropical and subtropical waters (except the Mediterranean Sea) [22,23]. Although capable of long distance movements, conventional and archival tagging indicate regional fidelity for bigeye tuna to geographical points of attraction [24,25]. Catch data from surface gears [22] indicate that the main breeding and nursery area of Atlantic bigeye tuna is located in the Gulf of Guinea, whereas adult feeding grounds are placed at both northern and southern temperate areas (Fig. 1). The exact timing of spawning migrations towards equatorial areas, and whether the adults return to their original northern and southern feeding zones after spawning remain open questions.

Several studies [10,26-28], reported geographic heterogeneity of bigeye tuna mt lineages within and among oceans. Both mt sequence and RFLP data revealed the existence of two highly divergent groups: Clade I that is present both in the Atlantic Ocean and Indo-Pacific Ocean, and Clade II that is almost exclusive to the Atlantic Ocean [10,26-28]. The origin of the two mt clades has been related to temperature fluctuations during the Pleistocene [29] that temporarily impeded bigeye tuna migration around the Cape of Good Hope [26]. Afterwards, unidirectional gene flow of mt Clade I from the Indo-Pacific Ocean into the Atlantic Ocean (promoted by the strong Agulhas Current that flows westward off southern Africa) during an interglacial period would have resulted in secondary contact and the contemporary asymmetrical distribution of the mt clades [10,26-28].

The contemporary presence of individuals of both mt clades in sympatry across the Atlantic Ocean but not in the Indo-Pacific Ocean poses several interesting questions that require further investigation using nuclear data. If individuals of mt Clade I and Clade II show high genetic divergence at the nuclear level, the two lineages could represent distinct cryptic species. Alternatively, individuals from both mt clades could interbreed in the Atlantic Ocean, a process that would render homogeneity at the nuclear level. On the other hand, the presence of individuals of mt Clade I in both the Atlantic Ocean and Indo-Pacific Ocean allows testing whether gene flow around the Cape of Good Hope is still occurring at present within this clade, as well as the directionality of the process.

Besides the above-mentioned historical factors, contemporary ecological factors such as e.g. phylopatric behaviour of adults could play an important role in shaping the current genetic structure of bigeye tuna. In order to discern the relative role of historical and ecological factors, the comparison of molecular markers of mt and nuclear origin is particularly informative. For instance, discordance between mt and nuclear markers in describing population structure have been used to asses sex-biased dispersal or philopatry [30-32]. In the case of the bigeye tuna, the comparison of allele-size frequencies at one mt and four nuclear loci in individuals from the Atlantic Ocean and Indo-Pacific Ocean tentatively rejected the hypothesis that the two mt clades could correspond to two cryptic species [33]. However, the population genetic analyses were not conclusive regarding gene flow around the Cape of Good Hope since allele frequencies of two nuclear loci supported the hypothesis of gene flow disruption between the Atlantic Ocean and the Indo-Pacific Ocean whereas those of another two nuclear loci failed to support the potential genetic break [33].

In this study, we gathered the most comprehensive genetic data set on Atlantic bigeye tuna to date. We analyze allele size variation of nine polymorphic microsatellite loci in four Atlantic locations that were surveyed in a previous study [10], and analyzed using mitochondrial control region sequence data. For a comparison of gene flow between ocean basins, we added one sample from the Pacific Ocean and another from the Indian Ocean. Microsatellite allele size data were analyzed with a variety of population genetic methods including hierarchical *F*-statistics, Mantel tests to determine for isolation by distance, Bayesian estimation of the number of populations, and coalescence-based estimation of the magnitude of gene flow. The main goal of these population genetic analyses was to determine genetic structure of bigeye tuna within the Atlantic Ocean based on nuclear data, and to compare it with mitochondrial evidence. We also tested the null hypothesis of genetic homogeneity (i.e. panmixia) worldwide, and estimated the magnitude and direction of gene flow between the Atlantic Ocean, and the Indo-Pacific region.

## Results

### Temporal genetic stability within Atlantic bigeye tuna populations

Three locations in the Atlantic Ocean, namely Canada, Canary islands, and Guinea were sampled in two different years (2001 and 2003) in order to test temporal stability of genetic variability. After Bonferroni correction, 12 out of 54 exact tests remained significant, which indicates their departure from Hardy-Weinberg equilibrium (HWE) (Table 1). Of these, only one locus exact test (TA161 at Guinea2) showed heterozygote excess characterized by a negative *F*_*IS *_value. According to the geographical origin of the samples, most (83%) of *F*_*IS *_significant values were found to belong to Guinea and Guinea 2 sampling sites (Table 1). To test for differences of *F*_*IS *_values between different years and loci, we performed a repeated measure ANOVA with year and locus as repeated measures. Both year and locus were modeled as random factors. The selection of the final model was based on the Akaike Information Criterion (AIC) [34]. Two models were considered significantly different when their AIC difference was larger than 2 (AIC ≥ 2.0) [35]. The best fitting model obtained (lowest AIC and significant ΔAIC ≥ 2.5) included the factor locus only, whereas year and the interaction between year and locus were not significant (ΔAIC ≤ 1.7). Furthermore, the analyses of the *F*_*ST *_and *R*_*ST *_pairwise comparisons between first and second year replicate comparisons at Guinea, Canary Islands, and Canada were each not significant (Table 2).

**Table 1:** Summary statistics for nine microsatellite loci of Atlantic bigeye tuna populations *

		Locus									
	
Population		TA102	TA113	TA117	TA121	TA161	TA208	TTH4	TTH208	TTH217	Mean all population
Guinea	*N*	45	48	47	46	48	47	48	47	45	46.8
	*N*_*A*_	23	31	26	14	21	15	24	20	9	20.3
	*N*_*S*_	17.21	21.55	20.21	12.02	15.86	10.95	18.52	16.03	7.30	15.52
	*H*_*E*_	0.929	0.952	0.951	0.900	0.929	0.852	0.944	0.925	0.738	0.902
	*Ho*	0.800	0.896	0.872	0.910	0.813	0.894	0.917	0.830	0.711	0.849
	*F*_*IS*_	**0.140**	**0.060**	**0.083**	0.093	0.127	-0.049	0.029	0.103	0.037	0.071
Guinea2	*N*	47	50	50	50	50	50	50	50	49	49.5
	*N*_*A*_	23	27	24	16	14	13	26	19	8	18.9
	*N*_*S*_	15.62	20.40	19.37	13.15	14.79	10.47	18.67	15.36	6.89	14.97
	*H*_*E*_	0.912	0.957	0.950	0.899	0.929	0.876	0.942	0.932	0.750	0.905
	*Ho*	0.766	0.900	0.820	0.908	0.980	0.880	0.860	0.780	0.633	0.836
	*F*_*IS*_	**0.162**	**0.061**	**0.138**	-0.014	**-0.056**	-0.004	**0.087**	**0.165**	0.158	0.076
Azores	*N*	49	50	50	50	50	50	47	49	49	49.3
	*N*_*A*_	21	30	25	15	20	16	21	21	10	19.9
	*N*_*S*_	16.09	21.58	18.65	12.82	15.62	11.33	17.44	16.66	7.85	15.34
	*H*_*E*_	0.924	0.960	0.949	0.906	0.929	0.866	0.946	0.925	0.776	0.909
	*Ho*	0.755	0.900	1.000	0.915	0.800	0.820	0.830	0.816	0.755	0.843
	*F*_*IS*_	0.184	0.063	-0.054	0.082	0.140	0.054	0.124	0.118	0.028	0.082
Canary	*N*	32	33	33	33	33	32	29	33	33	32.3
	*N*_*A*_	21	30	20	17	18	14	24	17	6	18.5
	*N*_*S*_	18.27	23.65	17.24	14.53	15.39	12.00	19.91	15.25	5.51	15.75
	*H*_*E*_	0.948	0.964	0.943	0.905	0.931	0.878	0.915	0.921	0.668	0.897
	*Ho*	0.875	0.879	0.939	0.919	0.909	0.906	0.862	0.818	0.636	0.860
	*F*_*IS*_	0.078	0.090	0.004	0.145	0.023	-0.033	0.059	0.113	0.048	0.060
Canary2	*N*	31	32	32	32	31	31	28	31	31	31
	*N*_*A*_	20	27	24	16	16	13	22	20	9	18.5
	*N*_*S*_	17.46	23.00	20.39	14.10	14.31	11.79	19.56	16.61	7.89	16.12
	*H*_*E*_	0.936	0.967	0.952	0.884	0.916	0.887	0.955	0.924	0.753	0.908
	*Ho*	0.710	0.875	0.969	0.898	0.871	0.807	0.750	0.839	0.645	0.818
	*F*_*IS*_	0.245	0.096	-0.017	0.132	0.050	0.093	**0.217**	0.094	0.145	0.115
Canada	*N*	40	40	40	40	40	40	40	40	40	40
	*N*_*A*_	19	31	22	17	18	13	26	19	9	19.3
	*N*_*S*_	15.40	23.44	18.31	13.97	15.21	10.56	19.72	15.63	7.25	15.5
	*H*_*E*_	0.917	0.963	0.936	0.900	0.914	0.867	0.949	0.930	0.773	0.905
	*Ho*	0.775	0.925	0.975	0.912	0.900	0.850	0.875	0.725	0.625	0.840
	*F*_*IS*_	0.156	0.040	-0.042	0.068	0.016	0.020	0.079	0.222	0.193	0.082
Canada2	*N*	23	23	23	23	23	23	21	23	23	22.8
	*N*_*A*_	20	21	17	14	14	12	17	16	8	15.4
	*N*_*S*_	19.02	20.10	16.36	13.47	13.55	11.56	17.00	15.30	7.82	14.91
	*H*_*E*_	0.934	0.940	0.896	0.854	0.901	0.885	0.940	0.905	0.825	0.898
	*Ho*	0.696	0.913	0.739	0.873	0.826	0.913	0.810	0.870	0.826	0.829
	*F*_*IS*_	0.260	0.029	0.178	-0.046	0.085	-0.032	0.141	0.040	-0.001	0.074
Indian	*N*	44	46	46	46	46	46	46	46	46	45.8
	*N*_*A*_	20	29	21	17	21	16	25	22	8	19.9
	*N*_*S*_	16.13	21.87	17.44	13.85	17.11	12.61	18.82	16.78	6.30	15.66
	*H*_*E*_	0.932	0.961	0.940	0.904	0.940	0.843	0.949	0.925	0.701	0.899
	*Ho*	0.955	0.935	0.870	0.914	0.804	0.783	0.935	0.587	0.609	0.821
	*F*_*IS*_	-0.024	0.027	0.076	0.001	0.146	0.072	0.015	**0.368**	0.133	0.090
Pacific	*N*	39	49	49	49	49	49	46	47	49	47.3
	*N*_*A*_	18	30	23	18	20	16	25	20	11	20.1
	*N*_*S*_	14.45	21.01	17.05	14.25	16.40	10.22	19.29	15.51	7.67	15.09
	*H*_*E*_	0.902	0.956	0.936	0.914	0.942	0.773	0.950	0.925	0.711	0.890
	*Ho*	0.843	0.898	0.939	0.923	0.939	0.714	0.848	0.702	0.633	0.826
	*F*_*IS*_	0.063	0.061	-0.003	-0.039	0.003	0.077	0.108	**0.243**	0.111	0.067
Mean all loci	*N*	38.9	41.22	41.1	41	41.1	40.9	39.4	40.7	40.5	40.5
	*N*_*A*_	20.5	28.4	22.4	16	18	14.2	23.3	19.3	8.7	19
	*N*_*S*_	16.63	21.84	18.33	13.57	15.36	11.28	18.77	15.90	7.16	15.43
	*H*_*E*_	0.926	0.958	0.939	0.896	0.926	0.858	0.943	0.923	0.744	0.901
	*Ho*	0.797	0.902	0.902	0.908	0.871	0.841	0.854	0.774	0.675	0.742
	*F*_*IS*_	0.133	0.058	0.045	0.045	0.060	0.024	0.088	0.174	0.098	0.080

* *N *= sample size, *N*_*A *_= number of alleles per locus, *N*_*S *_= number of alleles per locus standarized to the smallest sample size (23), expected (*H*_*E*_) and observed (*Ho*) heterozygosities, *F*_*IS *_= Wright's statistics. Bold *F*_*IS *_values are significant probability estimates after Bonferroni correction [83]

**Table 2:** Multilocus estimates for *F*_*ST *_(below diagonal) and *R*_*ST *_(above diagonal) between sample pairs from nine microsatellite loci in Atlantic bigeye tuna*.

	Guinea	Guinea 2	Azores	Canary	Canary 2	Canada	Canada 2	Indian	Pacific
Guinea	--	0,003	0,001	0,010	0,000	0,007	0,004	0,019	0,014
Guinea 2	0.005	--	0,001	0,012	0,000	0,006	0,002	0,013	0,017
Azores	0.002	0.000	--	0,000	0,004	0,001	0,011	0,006	0,008
Canary	0.002	0.005	0.002	--	0,002	0,017	0,002	0,004	0,007
Canary 2	0.004	0.001	0.001	0.004	--	0,002	0,004	0,002	0,000
Canada	0.001	0.008	0.001	0.008	0.002	--	0,003	0,020	0,017
Canada 2	0.006	0.012	0.009	**0.018**	0.011	0.001	--	0,003	0,014
Indian	**0.010**	**0.011**	0.005	0.009	0.005	**0.010**	**0.020**	--	0,002
Pacific	**0.011**	**0.011**	0.007	0.009	0.009	**0.014**	**0.027**	0.000	--

*Bold *P *values are significant after sequential Bonferroni correction for 36 multiple tests (*P *< 0.0014).

### Genetic variability and population structure among Atlantic bigeye tuna population samples

In order to characterize population genetic variation among Atlantic bigeye tuna, analyses were performed based on four Atlantic Ocean locations (the above mentioned three plus Azores), and included also one Indian Ocean, and one Pacific Ocean locations as outgroups. Genetic analyses were based only on those individuals that were correctly genotyped for at least seven loci (only nine out of 380 samples did not met this requirement and were excluded). The amount of genetic variability, in terms of average number of alleles (*N*_*S*_) and observed heterozygosity was similar among sampling sites for the same microsatellite locus (Table 1). However, there were large differences among loci, with values of average observed heterozygosity per locus ranging from *H*_*T *_= 0.675 at locus TTH217 to *H*_*T *_= 0.908 at locus TA121. Overall, the average observed heterozygosity per locus and population was high (*H*_*T *_= 0.742). The total *N*_*S *_varied from 7.16 (for TTH217) to 18.77 (TTH4) (Table 1). After Bonferroni correction, 7 out of 54 locus exact tests remained significant, which supports their departure from HWE. All showed significant heterozygote deficits in three (locus TTH208), and one (loci TA102, TA113, TA117, and TA161) sampling sites. Only locus TA161 deviated from HWE due to null alleles as detected using a null allele test based on expected homozygote and heterozygote allele size difference frequencies [36]. Since taking this locus out of the analyses did not qualitatively affect population comparisons (data not shown), it was included in further analyses. Tests for linkage disequilibrium did not show any significant value for any of the comparisons.

**Figure 2:**
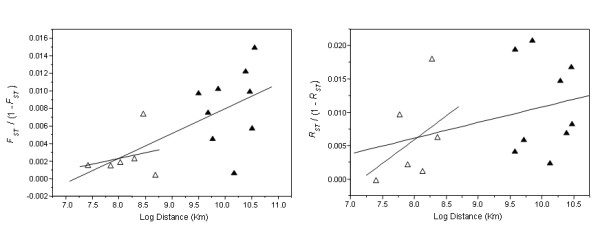
**Genetic isolation by distance in bigeye tuna inferred from multilocus estimates of *F*_*ST *_and *R*_*ST *_versus geographical distance.** The pairwise comparisons involved samples of Atlantic Ocean comparisons (open triangles), and comparisons between samples of Atlantic Ocean and the Indo-Pacific region (solid triangles).

The allele size permutation test rendered non-significant differences between *F*_*ST *_and *R*_*ST *_estimates (*P *= 0.3). Multilocus pairwise estimates of *F*_*ST *_showed nine significant comparisons after Bonferroni correction (Table 2). Pacific and Indian Ocean pairwise comparisons with Guinea and Canada sampling sites, but not with Azores and Canary Islands, were each significant. Within Atlantic Ocean comparisons were generally not significant. None of the *R*_*ST *_pairwise comparisons were significant after Bonferroni correction (Table 2). According to a Mantel test, we found no significant correlation between genetic and geographical distances for both Atlantic Ocean samples or across the entire studied geographic range when using *F*_*ST *_(R^2 ^= 0.23, *P *= 0.71; R^2 ^= 0.66, *P *= 0.99, respectively) or *R*_*ST *_(R^2 ^= 0.23, *P *= 0.71; R^2 ^= 0.66, *P *= 0.99) estimates (Fig. 2).

The hierarchical AMOVA revealed overall significant genetic structuring of the analyzed samples (Table 3). Most of the total genetic variance was found within populations. The hypothesis that nuclear variation could be structured according to mt clades was clearly rejected (Table 3). Geographic structuring of nuclear variation according to ocean basins (Atlantic Ocean, Indian Ocean, and Pacific Ocean) was also not supported by the AMOVA (Table 3), even when the Indian Ocean and the Pacific Ocean were grouped together (Indo-Pacific region) an analyzed against the Atlantic Ocean (result not shown).

**Table 3:** Analisis of molecular variance (AMOVA) of temporal and spatial genetic variation in Atlantic bigeye tuna for nine microsatellites*.

Structure tested	Variance	% total	*F Statistics*	*P*
1. One group (Guinea, Azores, Canary, Canada, Indian, Pacific)				
Among populations	0.019	0.53	*F*_*ST *_= 0.007	**0.00**
Within populations	3.660	99.47		
2. Two groups Clade I (Guinea, Azores, Canary, Canada, Indian, Pacific) vs. Clade II (Guinea, Azores, Canary, Canada)				
Among groups	0.007	0.17	*F*_*CT *_= 0.002	0.17
Within groups	0.015	0.39	*F*_*SC *_= 0.004	**0.04**
Within populations	3.952	99.44	*F*_*ST *_= 0.005	**0.00**
3. Three groups (Guinea, Azores, Canary, Canada) vs. (Indian Ocean) vs. (Pacific Ocean)				
Among groups	0.020	0.55	*F*_*CT *_= 0.006	0.07
Within groups	0.009	0.25	*F*_*SC *_= 0.003	**0.05**
Within populations	3.520	99.2	*F*_*ST *_= 0.008	**0.00**

*Bold *P *numbers are significant values.

**Figure 3:**
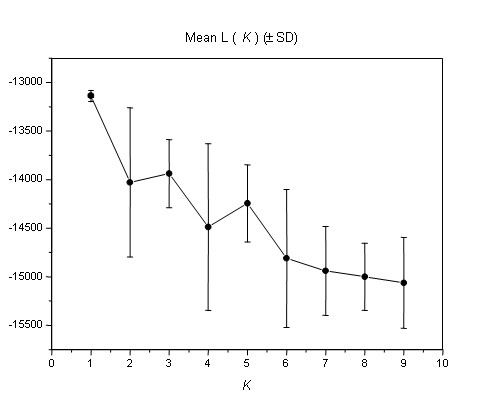
Number of bigeye tuna populations with the highest posterior probability expressed as the mean likelihood (log P (*X*|*K*)), over 20 runs for each *K *(one to nine).

The number of populations (and the assignment of individuals to each population) was estimated using Bayesian inferences. Although the prior parameters for the *F *model (gamma distribution with mean 0.01 and standard deviation 0.05) were chosen to allow the existence of two possible populations with very similar allele frequencies [37], the highest posterior probability value was found at *K *= 1 (Fig. 3). If the latent number of populations was not pre-specified [38], the highest posterior probability was also found for a partition of one. A Bayesian inference of jointly the probability of assignment of individuals to populations and the number of populations [39,40] estimated that the number of populations with the highest posterior probability was also for *K *= 1 (*P *= 0.93).

### Effective population size and migration rates

Migration rates and effective population size of bigeye tuna populations were estimated based on geographic regions (ocean basins) of origin using both ML and BI methods [20]. The ML inferences did not return reliable confidence intervals using profile likelihoods even for very long runs (not shown), but none of the runs contradicted the Bayesian analysis. The results from the Bayesian inference were more stable and reliable. Estimates of the mutation scaled population size parameter were higher in the Atlantic Ocean (Θ = 6.07) than in the Indian Ocean (Θ = 2.33) and the Pacific Ocean (Θ = 1.67). These estimates were translated to an average effective population size (*Ne*) of 15175, 5825, and 4175 bigeye tuna individuals for the Atlantic Ocean, the Indian Ocean, and the Pacific Ocean, respectively (assuming a microsatellite mutation rate of 10^-4 ^per locus per generation; [41]). Marked differences in ratios of effective population size (mean *Ne *= 8391.67 ± 5932.19) to census population size (as derived from annual catch data, and averaging 403.000 tones for bigeye tuna; [42]) are commonplace in marine fishes [43,44], and can be explained either by historical events such as e.g. past population bottlenecks or by life history traits such as e.g. strong bias in reproductive success or size-dependent fecundity [44-46]. In order to discern among both competing hypotheses, we calculated values of *M *(a statistic that estimates population bottleneck history using the ratio of the number of alleles to the range in allele size; [47]), which were above the theoretical threshold of 0.68, indicating that none of the Atlantic, Indian and Pacific Ocean populations has experienced recent population reduction. Hence, it is likely that the low *Ne *observed in bigeye tuna may be due to life-history traits as previously reported for other marine fishes [44].

**Figure 4:**
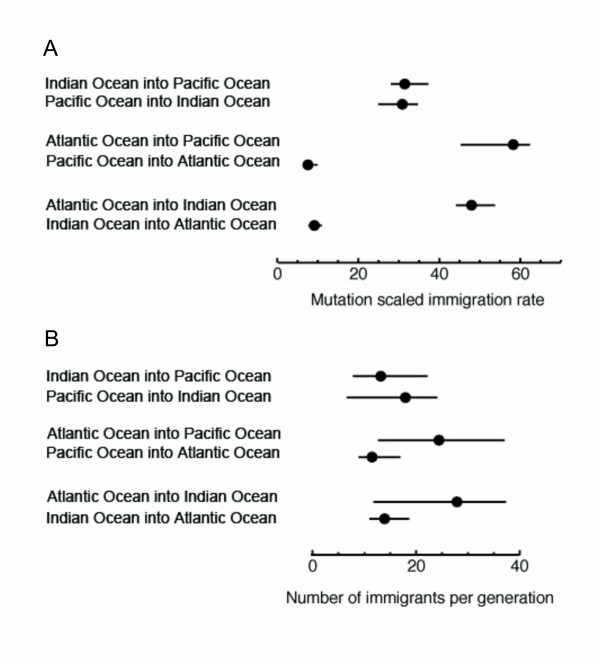
**Estimates of gene flow for bigeye tuna populations among ocean basins based on Bayesian inferences of migration rates and population sizes.** Dots are the maximum posterior values, and the lines show the 95% credibility intervals. (A) Mutation scaled immigration rate, *M*, between ocean basins. *M *is the ratio of immigration rate and mutation rate. (B) Number of immigrants per generation, *N*_m_. 95% confidence intervals are shown.

Migration rates seem to be symmetric between the Indian Ocean and the Pacific Ocean (Fig. 4A). However, immigration from and into the Atlantic Ocean seems to be highly asymmetrical with the Atlantic population providing twice as many immigrants into the Pacific Ocean and the Indian Ocean than those inferred between the Pacific Ocean and the Indian Ocean (Fig. 4A). Furthermore, immigration into the Atlantic Ocean showed the lowest rates. A likelihood ratio test revealed that these results were incompatible with symmetrical scaled immigration rates or with a common rate for all directions (P < 0.00001). Inferred numbers of immigrants per generation between ocean basins ranged between 11.5 and 27.9, Fig. 4B).

## Discussion

### Microsatellite data and HWE departures

Despite cross-specific PCR amplification, all analyzed microsatellites showed relatively high levels of polymorphism. Simulations using various combinations of effective population size (*N*_*e*_) and time since divergence (*t*) showed that these loci provided enough statistical power [48] for the detection of genetic variability in Atlantic bigeye tuna. In fact, previously loci TA102, TA113, TA121, and TA161 were applied to analyze population genetic structuring of bigeye tuna in the Indian Ocean [49], and locus TA161 was able to detect weak genetic differentiation in the Pacific Ocean [28]. Significant deviations from HWE were found in few populations at five loci, which were different to those showing departures from HWE in *Thunnus albacares *[50]. The majority of the detected departures from HWE were due to significant heterozygote deficiency. Most of the significant *F*_*IS *_locus-population comparisons involved Guinea samples of different years versus other Atlantic populations, being deviations attributed to homozygote excess. According to the ANOVA test only the genetic factor (the locus) and neither the time nor the combination of both factors was responsible for the deviation from HWE. On the other hand, the only case of departure from HWE due to null alleles was associated to the Guinea location. All these results together highlight the singularity of the Guinea location, and could be explained in terms of admixture of genetically distinct cohorts (Whalund effect) within this site, owing to the fact that the Gulf of Guinea is known as the main spawning and nursery area for bigeye tuna in the Atlantic Ocean [22,51]. Similar patterns of HWE departures have been associated with spawning or nursery populations in the Atlantic bluefin tuna (*Thunnus thynnus*) [52], and lemon shark (*Negaprion brevirostris*) [53].

### Genetic variation and population structure of Atlantic bigeye tuna

High values of genetic variability (mean number of alleles per locus and heterozygosity) characterized all studied locations. These values are similar to those previously reported for other scombroid fishes, such as the bluefin tuna [52,54], yellowfin tuna [50] and bigeye tuna [49,55], and consistent with the average for marine fishes [56].

Within the Atlantic Ocean, no significant genetic differentiation between temporal replicates or geographic locations was observed (all *F*_*ST *_and *R*_*ST *_pairwise comparisons rendered low and non-significant values except a *F*_*ST *_pairwise comparison between Canada2 and Canary). Additionally, the Mantel test failed to detect significant correlation between geographical and genetic distances, which discarded isolation by distance among populations within the Atlantic Ocean. Overall, these results support unrestricted gene flow among bigeye tuna populations within the Atlantic Ocean. Similarly, no genetic structuring was reported for bigeye tuna within the Pacific Ocean [28] nor within the Indian Ocean [49]. The lack of genetic differentiation within each ocean basin may not be that surprising given the relatively high dispersal capability of bigeye tunas [24]. Nevertheless, some statistical tests and analyzed loci have shown weak evidence for small genetic differences between most separated locations in the Pacific, suggesting that isolation by distance might be promoting subtle differentiation among populations, which could be at present difficult to document with the current loci and sample sizes [28]

The hypothesis of a single panmictic unit for bigeye tuna worldwide is supported by microsatellite data based on the lack of significant differentiation in between-ocean *R*_*ST *_comparisons, and the comparatively low *F*_*ST *_and *R*_*ST *_values obtained. These values are by far lower than the mean *F*_*ST *_considered for marine fishes (0.062, [57-59]), but on the same order as those reported in other studies where range-wide panmixia was suggested [60-63]. Hierarchical AMOVA, Mantel test, as well as clustering and assignment tests rendered consistent results, and also supported the hypothesis of no genetic structure for tuna populations at an inter-oceanic scale.

Therefore, nuclear and mt evidence are clearly discordant with respect to inter-oceanic genetic differentiation. While the former, as mentioned above, fails to support any genetic structuring, the latter supports genetic differentiation between Atlantic Ocean and Indo-Pacific region bigeye tuna populations based on pairwise *Φ*_*ST *_comparisons [10]. Strong phylogeographic association of mtDNA (two clades), compared to microsatellites, has also been previously documented in e.g. blue marlin [64]. In the bigeye tuna, the main source of disagreement largely arises from the existence in the Atlantic Ocean of two highly divergent mt clades [10,26,27], which were not recovered by AMOVA tests based on nuclear data. The origin of the two mt clades (I and II) has been proposed to be associated with past genetic isolation of bigeye tuna Atlantic Ocean populations from those of the Indo-Pacific region during Pleistocene glacial maxima [10,26-28]. Once temperatures rose during inter-glacial periods, gene flow could be re-established through the Cape of Good Hope. The present-day observed pattern would have been achieved when individuals belonging to mt Clade I entered the Atlantic Ocean from the Indo-Pacific region, whereas individuals belonging to mt Clade II apparently remained within the Atlantic Ocean. Microsatellite data indicate that despite the accumulated high sequence mt divergences, individuals from both clades were not reproductively isolated and thus, secondary contact in the Atlantic Ocean led to interbreeding, and present-day genetic homogenization at the nuclear level [33].

### Migration patterns of bigeye tuna populations

Bayesian inference of migration rates indicates gene flow rates of the same order of magnitude inferred for other pelagic fishes such as e.g. sardines [65]. There is migration in both directions between the Pacific Ocean and the Indian Ocean, likely facilitated by both the Indonesian Ocean Current that flows from the Pacific Ocean into the Indian Ocean, as well as the seasonal reversal of this current [66]. However, according to our results, the main trans-oceanic migratory activity is occurring around the Cape of Good Hope. The region off South Africa is both topographically and hydrologically complex [29], which complicates understanding of bigeye tuna migration patterns. At both sides of the Cape of Good Hope, the South Atlantic Ocean [67] and the southwest Indian Ocean [68] gyres are connected by the strong Agulhas Current that flows from the Indian Ocean into the Atlantic Ocean [69]. As the Agulhas Current reaches the southern tip of the continental shelf of Africa, it turns almost completely back on itself, and flows eastward as the Agulhas retroflection or return current [69,70]. According to our results (Fig. 4A), immigration from the Atlantic Ocean into the Indo-Pacific region doubles that inferred between the Indian Ocean and the Pacific Ocean whereas scaled immigration rates into the Atlantic Ocean are the lowest, suggesting that immigration is less important for the maintenance of variability in the Atlantic Ocean than in other ocean basins.

Our results indicate that the actual number of individuals moving per generation (Fig. 4B) is several orders of magnitude lower than bigeye population sizes in each ocean (millions of individuals). Thus, the possibility of detecting changes in mt clade proportions in each ocean through catch statistics is extremely small. In fact, the proportions of individuals of mt Clade I (about 25%) and mt Clade II (about 75%) in the studied populations of Atlantic bigeye tuna seemed to remain constant over the years (Fig. 1, [10]) and virtually no individuals of mt Clade II have been reported from the Indian Ocean [49]. Moreover, significant allele-frequency differences at one intronic locus and one microsatellite locus were found between Atlantic and Indo-Pacific samples, supporting simple mixture but little movement of bigeye tuna individuals around the Cape of Good Hope [33]. Thus, it seems that the main invasion of individuals from the Indo-Pacific region carrying mt Clade I haplotypes into the Atlantic Ocean may have occurred during an earlier warmer inter-glacial period rather than after the last glacial maximum.

Alternatively, lack of nuclear differentiation coupled with strong mt divergences could also indicate instances of male-biased dispersal (females would exhibit phylopatric behavior and thus would be considered sedentary with regards to the ocean basin) [33]. Although, the existence of independent spawning areas in each ocean basin [26] may support the existence of homing behavior in bigeye tuna, and there are reports of regional fidelity in bigeye tuna [24], the existence of homing behavior remains an open question, and tagging data showing male trans-oceanic displacements is required to understand current gene flow among bigeye tuna populations.

Finally, the inferred asymmetrical immigration pattern out and into the Atlantic Ocean could simply reflect the presence in this ocean basin of Clade II, which is not shared with the Indo-Pacific region. The number of immigrants into the Atlantic Ocean could be less extreme because of the presence of the two clades in the Atlantic Ocean, which would produce larger variability, and thus, larger effective population sizes. However, it is important to note here that the inferred migration rates may also reflect a sampling bias since the data set is allele-rich and the present study only includes samples from one Indian Ocean and one Pacific Ocean location. Therefore, these two ocean basins may be underrepresented, and the estimates of the number of immigrants per generation (*N*_m_) have a large variance associated (Fig. 4B).

### F_ST _as a measure of genetic structure and gene flow

**Figure 5:**
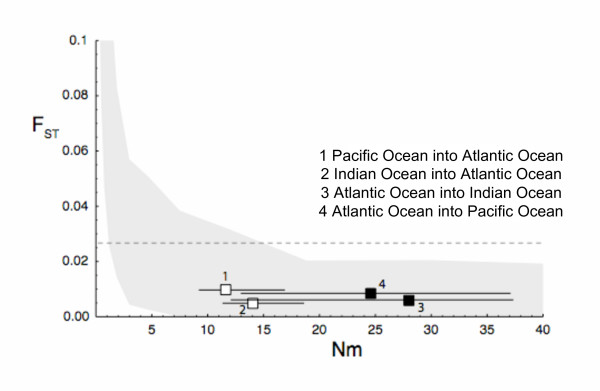
***F***_**ST **_**values estimated from simulated data.** The X-axis shows the number of immigrants per generation *N*_m _that was used to simulate 100 data sets for 20 equidistant points along the X-axis. The gray area covers the 95% confidence area. The filled and open squares show from and into Atlantic Ocean comparisons, respectively. 95% confidence intervals are shown. The dotted line shows the maximal *F*_ST _value detected (corresponding to Canada2 versus Pacific pairwise comparison)

Most population genetic analyses performed in this study suggest lack of population structure, and relatively high levels of gene flow among bigeye tuna populations at a global scale in agreement with other population genetic analyses. However, nine out of 36 *F*_*ST *_pairwise comparisons detected significant population structure, mostly involving comparisons between populations of the Atlantic Ocean and those of the Indo-Pacific region (Table 2). In order to understand this discrepancy, *F*_*ST *_analyses were performed using simulated data of three populations (Atlantic Ocean, Indian Ocean, and Pacific Ocean) (Figure 5). Estimates of *F*_*ST *_values from data with large number of immigrants (like the one estimated from bigeye tuna microsatellite data) showed large 95% confidence intervals that include *F*_*ST *_values up to about 0.2. The presumed differentiation was most likely an artifact of the large number of alleles present in the sample. Although *F*_*ST *_statistics [71] are widely used for describing population genetic structure, they present some limitations, including the implicit assumption of uniform effective population sizes, and symmetric migration rates. Violation of these assumptions is particularly worrisome when using highly polymorphic molecular markers with high mutation rates (e.g. microsatellites) to analyze weakly structured populations [72] with large effective population sizes [56,73,74]. *F*_*ST *_estimations assume that there is no error in the sample frequencies, but with many alleles per locus the precision of sample frequencies is questionable. In such cases, the use of both *R*_*ST*_, which is independent of the mutation rate [75,76], and coalescent methods, which use sample frequencies and not the population frequencies render more accurate and reliable estimates of genetic differentiation than *F*_*ST*_.

## Conclusion

Population genetic analyses based on microsatellite data of bigeye tuna populations showed unrestricted gene flow within the Atlantic Ocean, and supported the existence of a spawning and nursery region in the Gulf of Guinea. At a global scale, *R*_*ST *_statistics, Bayesian cluster analyses, and coalescence-based migration rate inferences based on nuclear markers supported lack of trans-oceanic genetic structure, which contrasts with previously detected significant mt divergence of bigeye tuna between Atlantic Ocean and Indo-Pacific region due to the existence of two clades, one restricted to the Atlantic Ocean. Our results support interbreeding between individuals belonging to different clades in the Atlantic Ocean. Given the current distribution of bigeye tuna individuals belonging to the two clades, and the inferred asymmetrical nuclear migration rates between the Atlantic Ocean and the Indo-Pacific region, it is likely that little number of individuals (compare to the actual population sizes in each ocean) may currently move between the Atlantic Ocean and the Indo-Pacific region, maintaining the proportions of the mt clades in both oceans. Therefore, the current presence of individuals belonging to mt Clade I would have originated in an earlier interglacial period.

## Methods

### Sampling

A total of 380 individuals of bigeye tuna (*Thunnus obesus*) were obtained from different locations in the Atlantic Ocean (Gulf of Guinea, *N *= 50; Canary Islands, *N *= 34; Azores, *N *= 51 and Canada, *N *= 41), the Indian Ocean (*N *= 49), and the Pacific Ocean (*N *= 50) (Fig. 1). Three Atlantic Ocean sites (Guinea2, *N *= 50; Canary2, *N *= 32 and Canada 2, *N *= 23) were re-sampled a second year to investigate temporal stability of genetic structure in these regions (Fig. 1). Collections included exclusively juveniles except in Canada where adults were also captured (See Table 1[10]). ICCAT (International Commission for the Conservation of Atlantic tuna) collected Atlantic Ocean samples, IATTC (Inter-American Tropical Tuna Commission) provided Pacific Ocean samples, and a commercial fishing operator caught the Indian Ocean samples. Samples consisted of small pieces of muscle preserved either in absolute ethanol or frozen at -20°C.

### Microsatellite loci amplification and scoring

Genomic DNA was extracted from muscle tissue as previously described [10]. After optimizing reaction conditions, three microsatellite loci (TTH4, TTH208 and TTH217) originally isolated from *T. thynnus *[54], plus six microsatellite loci (TA102, TA113, TA117, TA121, TA161 and TA208) originally characterized from *T. albacares *[50], and already used in bigeye tuna [49,55] were PCR amplified from the different samples. PCR amplifications consisted of 35 cycles of denaturing at 95°C for 45 s, annealing at 60–65°C for 45 s, and extending at 72°C for 1 min. Cycles were followed by a final extension at 72°C for 10 min. PCRs contained approximately 5 ng of sample DNA, 0.1 U of Taq DNA polymerase (Eppendorf), 0.5 μM of each primer, 200 μM of each dNTP, one mM MgCl_2 _and 1xTaq buffer (Tris-HCl 67 mM, pH 8.3, MgCl_2 _1.5 mM), in a total volume of 10 μl. Forward primers were labeled with fluorescent dye (Invitrogen), and amplified products were run for size detection on an ABI 3700 automated sequencer. Data collection and sizing of alleles were carried out using GeneScan v3.1.2 and Genotyper v3.1 software (Applied Biosystems). Approximately 10% of the samples were re-run to ensure repeatability in scoring.

### Descriptive statistics

For each locus and sampling site (including second-year samples), observed and expected heterozygosities [77], number of alleles (*N*_A_), and number of alleles standardized to those of the population with the smallest sample size (*N*_S_) [78] were calculated using both GENETIX 4.02 [79] and FSTAT 2.9.3 [80]. Similarly, *F*_IS _statistic estimations that detect deviations from HWE, and the linkage disequilibrium test were performed for each locus and sampling site using the program GENEPOP version3.3 [81]. Significance of both analyses was tested with a Markov chain Monte Carlo (MCMC) that was run for 1000 batches of 2000 iterations each, with the first 500 iterations discarded before sampling [82]. *P *values from multiple comparisons were Bonferroni corrected [83]. MICRO-CHECKER v2.23 [36] was used to explore the existence of null alleles, and to evaluate their impact on the estimation of genetic differentiation. POWSIM v1.2 [48] was used to estimate whether the dataset used for genetic population analysis provided enough statistical power for detecting significant genetic differentiation. The nine population sampled were used for testing allele frequency homogeneity at nine locus separately and combining information for the multiple loci using Fisher's exact and traditional chi-squared tests. Simulations were run using various combinations of *N*_*e *_and *t *(where *N*_*e *_is the effective population size and t is the time since divergence, respectively), leading to *F*_*ST *_of 0.001 and 0.0025, which reflects the magnitude of *F*_*ST *_and *N*_*e *_values estimated from our empirical data. We also estimated the α error (type I) by performing a simulation of no divergence among samples (i.e. setting *t *= 0 that leads a value of *F*_*ST *_= 0). Results indicated that the probability for detecting population structure were high and statistically significant, corresponding to 76% and 72% for the *N*_*e*_/*t *combinations of 9,000/18 and 10,000/20 and 100% for both the *N*_*e*_/*t *combinations of 9,000/45 and 10,000/45, respectively. When *F*_*ST *_was set to zero, the proportion of false significances (α) was 1%, which is lower to the intended value of 5%.

### Classic measures of genetic differentiation

A possible geographical pattern in the distribution of genetic variability was tested for each pair of sampling sites (36 pairwise comparisons including second-year samples) using *F*_*ST *_(infinite-allele model (IAM); [84] and *R*_*ST *_(stepwise mutation model (SMM); [85] pairwise comparisons using ARLEQUIN v3.0 [86] and RST-CALC [87], respectively. In addition, individuals were grouped based on their mitochondrial affiliation (Clade I or II), as well as based on ocean basins (Atlantic Ocean, Indian Ocean, or Pacific Ocean), and the corresponding pairwise *F*_*ST *_and *R*_*ST *_estimates were calculated. In all instances with multiple tests, *P *values were adjusted using the Bonferroni correction [83]. In order to test the null hypotheses of no contribution of stepwise mutation to genetic differentiation (*R*_*ST *_= *F*_*ST*_), a total of 1000 allele size permutations were computed using SPAGEDI 1.1 [88] to provide a simulated distribution of *R*_*ST *_values (ρ_*ST*_). Mantel test was used to test correlation between geographical and genetic distances as implemented in GENEPOP version3.3 [81]. The logarithm of geographical distance in kilometers was regressed against either *F*_*ST*_/(1 - *F*_*ST*_) or *R*_*ST*_/(1 - *R*_*ST*_) using the program ISOLDE in GENEPOP.

Hierarchical genetic structuring of microsatellite data (only first year sampling) was analyzed by assessing the relative contribution among groups, within groups and within population components for partitions of total molecular variance (AMOVA) [89] using ARLEQUIN v3.0 [86] and 20,000 permutations. We specifically tested the null hypothesis of panmixia, as well as of alternative structuring by mt clade (Clade I and Clade II) and by geographical region (Atlantic Ocean, Indian Ocean and Pacific Ocean).

### Analyses to establish the number of populations

In order to complement and contrast the results obtained with the classical standard *F*-statistics [90] we also inferred population structure using a Bayesian approach. The number of populations (*K*) with the highest posterior probability given the data (only first year sampling) was estimated using STRUCTURE [91], BAPS v3.1 [38] and STRUCTURAMA [92]. In STRUCTURE, we selected the admixture model and the option of correlated allele frequencies between populations (also called the *F*-model) because it is considered the superior model for detecting structure among closely related populations [37]. MCMC consisted on 1 × 10^5 ^burn-in iterations followed by 1,5 × 10^6 ^iterations. We explored *K *in the range of one and nine and we performed 20 runs for each *K *value. In BAPS [38], the Bayesian inference does not need the number of populations is pre-specified. We used the option "cluster groups of individuals" to run the program with default conditions. The program STRUCTURAMA [92] infers population genetic structure from genetic data by allowing the number of populations to be a random variable that follows a Dirichlet process prior [39,40]. We run 1 × 10^6 ^MCMC cycles, discarding the first 1 × 10^5 ^cycles as burn-in.

### Estimation of migration rates and effective population sizes

The program MIGRATE v 2.4 [93] was used to infer the population size parameter Θ (i.e. 4*N*_*e*_*μ*, where *N*_*e *_is the effective population size and *μ *is the mutation rate per site) and the migration rate, *M *(*M *= m/*μ*, where m is the immigration rate per generation) among bigeye tuna populations. These analyses used the Brownian mutation model [93] and mutation was considered to be constant for all loci. We used the Bayesian inference [20] and the maximum likelihood [17,18], modes. *F*_*ST *_estimates and a UPGMA tree were used as starting parameters for the estimation of Θ and *M*. For each locus in the data set, the ML was run for ten short and three long chains with 10,000 and 100,000-recorded genealogies, respectively, after discarding the first 10,000 genealogies (burn-in) for each chain. One of every 20 reconstructed genealogies was sampled for both the short and long chains. The Bayesian run consisted of one long chain with 50 millions-recorded parameter and genealogy changes after discarding the first 10,000 genealogies as burn-in for each locus. For all the analyses we used an adaptive heating scheme with 4 concurrent chains; the analyses were run on a cluster computer using up to 80 compute nodes. We conducted the analyses over two sets of data, the first one with microsatellite data structured into two groups equivalent to mt Clades I and II; and the second set of data structured according to geographical regions (Atlantic Ocean, Indian Ocean, and Pacific Ocean).

In order to test whether past populations bottlenecks could be responsible of the observed difference between census and effective population sizes, we used the M_P_val program to calculate M, a statistic that estimates population bottleneck history using the ratio of the number of alleles to the range in allele size [47].

### Simulated migration rates and F_ST _estimations

In order to better understand the limitations of *F*_*ST *_estimation, we explored the range of estimated *F*_*ST *_values for a range of migration rates. We simulated 20 times 100 datasets over the range of the number of immigrants per generation, *N*_*e*_*m *between 4 and 40 and report the 95% confidence intervals. The migration rate range covers the estimated migration rates of the real data using the coalescent inference method.

## Authors' contributions

EGG carried out the DNA genotyping, performed the statistical analyses and drafted the manuscript. PB undertook software implementation, statistical and simulation analyses as well as interpretation and discussion of the results. RZ conceived the study, supervised the genetic studies and analyses, and contributed to the writing of the manuscript. All authors read and approved the final manuscript.

## References

[B1] Avise JC Conservation genetics in the marine realm J Hered 1998 89 377 382 10.1093/jhered/89.5.377

[B2] Nesbø CL Rueness EK Iversen SA Skagen DW Jakobsen KS Phylogeography and population history of Atlantic mackerel (*Scomber scombrus *L.): a genealogical approach reveals genetic structuring among the eastern Atlantic stocks Proc R Soc Lond B 2000 267 281 292 10.1098/rspb.2000.0998 PMC1690521 10714883

[B3] Waples RS A multispecies approach to the analysis of gene flow in marine shore fishes Evolution 1987 41 385 400 10.2307/2409146 28568763

[B4] Zardoya R Castilho R Grande C Favre-Krey L Caetano S Marcato S Krey G Patarnello T Differential population structuring of two closely related fish species, the mackerel (*Scomber scombrus*) and the chub mackerel (*Scomber japonicus*), in the Mediterranean Sea Mol Ecol 2004 13 1785 1798 10.1111/j.1365-294X.2004.02198.x 15189203

[B5] Alvarado Bremer JR Vinas J Mejuto J Ely B Pla C Comparative phylogeography of Atlantic bluefin tuna and swordfish: the combined effects of vicariance, secondary contact, introgression, and population expansion on the regional phylogenies of two highly migratory pelagic fishes Mol Phylogenet Evol 2005 36 169 187 10.1016/j.ympev.2004.12.011 15904864

[B6] Hauser L Ward RD Carvalho GR Population identification in pelagic fish: the limits of molecular markers Advances in Molecular Ecology 1998 Amsterdam: IOS Press 191 224

[B7] Bohonak AJ Dispersal, gene flow, and population structure Q Rev Biol 1999 74 21 45 10.1086/392950 10081813

[B8] Whitlock BS McCauley DE Indirect measures of gene flow and migration. *F*_ST _≠ 1/(4Nm+1) Heredity 1999 82 117 125 10.1038/sj.hdy.6884960 10098262

[B9] Beerli P Gary R Carvalho Estimation of migration rates and population sizes in geographically structured populations Advances in Molecular Ecology. Series NS 1998 39 53

[B10] Martínez P Gonzalez EG Castilho R Zardoya R Genetic diversity and historical demography of Atlantic bigeye tuna (*Thunnus obesus*) Mol Phylogenet Evol 2006 39 404 416 10.1016/j.ympev.2005.07.022 16188460

[B11] Viñas J Alvarado Bremer J Pla C Phylogeography of the Atlantic bonito (*Sarda sarda*) in the northern Mediterranean: the combined effects of historical vicariance, population expansion, secondary invasion, and isolation by distance Mol Phylogenet Evol 2004 33 32 42 10.1016/j.ympev.2004.04.009 15324837

[B12] Pluzhnikov A Donnelly P Optimal sequencing strategies for surveying molecular genetic diversity Genetics 1996 144 1247 1262 8913765 10.1093/genetics/144.3.1247 PMC1207616

[B13] Felsenstein J Accuracy of coalescent likelihood estimates: Do we need more sites, more sequences, or more loci? Mol Biol Evol 2006 23 691 700 10.1093/molbev/msj079 16364968

[B14] Kingman JF Origins of the coalescent. 1974–1982 Genetics 2000 156 1461 1463 11102348 10.1093/genetics/156.4.1461 PMC1461350

[B15] Griffiths RC Tavare S Sampling theory for neutral alleles in a varying environment Philos Trans R Soc Lond B Biol Sci 1994 344 403 410 10.1098/rstb.1994.0079 7800710

[B16] Kuhner MK Yamato J Felsenstein J Estimating effective population size and mutation rate from sequence data using Metropolis-Hastings sampling Genetics 1995 140 1421 1430 7498781 10.1093/genetics/140.4.1421 PMC1206705

[B17] Beerli P Felsenstein J Maximum likelihood estimation of a migration matrix and effective population sizes in n subpopulations by using a coalescent approach Proc Natl Acad Sci USA 2001 98 4563 4568 10.1073/pnas.081068098 11287657 PMC31874

[B18] Beerli P Felsenstein J Maximum-likelihood estimation of migration rates and effective population numbers in two populations using a coalescent approach Genetics 1999 152 763 773 10353916 10.1093/genetics/152.2.763 PMC1460627

[B19] Beaumont MA Zhang W Balding DJ Approximate Bayesian computation in population genetics Genetics 2002 162 2025 2035 12524368 10.1093/genetics/162.4.2025 PMC1462356

[B20] Beerli P Comparison of Bayesian and maximum-likelihood inference of population genetic parameters Bioinformatics 2006 22 341 345 10.1093/bioinformatics/bti803 16317072

[B21] Drummond AJ Nicholls GK Rodrigo AG Solomon W Estimating mutation parameters, population history and genealogy simultaneously from temporally spaced sequence data Genetics 2002 161 1307 1320 12136032 10.1093/genetics/161.3.1307 PMC1462188

[B22] Fonteneau A Ariz J Delgado A Pallares P Pianet R A comparison of bigeye (*Thunnus obesus*) stocks and fisheries in the Atlantic, Indian and Pacific Oceans Col Vol Sci Pap ICCAT 2005 57 41 66

[B23] Hampton J Bigelow K Labelle M Effect of longline fishing depth, water temperature and dissolved oxygen on bigeye tuna (*Thunnus obesus*) abundance indices 1998 Community OFPSotP. B.P. D5, Noumea, New Caledonia 17

[B24] Schaefer KM Fuller DW Conventional and archival tagging of bigeye tuna (*Thunnus obesus*) in the eastern Equatorial Pacific Ocean Col Vol Sci Pap ICCAT 2005 57 67 84

[B25] Sibert JR Musyl MK Brill RW Horizontal movements of bigeye tuna (*Thunnus obesus*) near Hawaii determined by Kalman filter analysis of archival tagging data Fish Oceanogr 2003 12 141 151 10.1046/j.1365-2419.2003.00228.x

[B26] Alvarado Bremer JR Stequert B Robertson NW Ely B Genetic evidence for inter-oceanic subdivision of bigeye tuna (*Thunnus obesus*) populations Mar Biol 1998 132 547 557 10.1007/s002270050420

[B27] Chow S Okamoto H Miyabe N Hiramatsu K Genetic divergence between Atlantic and Indo-Pacific stocks of bigeye tuna (*Thunnus obesus*) and admixture around South Africa Mol Ecol 2000 9 221 227 10.1046/j.1365-294x.2000.00851.x 10672166

[B28] Grewe PM Hampton J An assessment of bigeye (*Thunnus obesus*) population structure in the Pacific Ocean, based on mitochondrial DNA and DNA microsatellite analysis Forum Fisheries Agency and Pelagic Fisheries Research Program: 1998 1998

[B29] Peeters FJC Acheson R Brummer GJA de Ruijter WPM Schneider RR Ganssen GM Ufkes E Kroon D Vigorous exchange between the Indian and Atlantic Oceans at the end of the past five glacial periods Nature 2004 430 661 665 10.1038/nature02785 15295596

[B30] Fraser DJ Lippe C Bernatchez L Consequences of unequal population size, asymmetric gene flow and sex-biased dispersal on population structure in brook charr (*Salvelinus fontinalis*) Mol Ecol 2004 13 67 80 10.1046/j.1365-294X.2003.02038.x 14653789

[B31] Lyrholm T Leimar O Johanneson B Gyllensten U Sex-biased dispersal in sperm whales: contrasting mitochondrial and nuclear genetic structure of global populations Proc Natl Acad Sci USA 1999 266 347 354 10.1098/rspb.1999.0644 PMC1689695 10097396

[B32] Shaw PW Arkhipkin AI Al-Khairulla H Genetic structuring of Patagonian toothfish populations in the Southwest Atlantic Ocean: the effect of the Antarctic Polar Front and deep-water troughs as barriers to genetic exchange Mol Ecol 2004 13 3293 3303 10.1111/j.1365-294X.2004.02327.x 15487990

[B33] Durand JD Collet A Chow S Guinand B Borsa P Nuclear and mitochondrial DNA markers indicate unidirectional gene flow of Indo-Pacific to Atlantic bigeye tuna (*Thunnus obesus*) populations, and their admixture off southern Africa Mar Biol 2005 147 313 322 10.1007/s00227-005-1564-2

[B34] Akaike H Csaksi BNPaF Information theory as an extension of the maximum likelihood principle2nd International Symposium on Information Theory 1973 Budapest, Hungary: Akademiai Kiado 267 281

[B35] Burnham KP Anderson DR Model Selection and Inference. A practical Informatio-theoretic Approach New York 1998

[B36] Van Oosterhout C Hutchinson WF Wills DPM Shipley P MICRO-CHECKER: software for identifying and correcting genotyping errors in microsatellite data Mol Ecol Notes 2004 4 535 538 10.1111/j.1471-8286.2004.00684.x

[B37] Falush D Stephens M Pritchard JK Inference of population structure using multilocus genotype data: linked loci and correlated allele frequencies Genetics 2003 164 1567 1587 12930761 10.1093/genetics/164.4.1567 PMC1462648

[B38] Corander J Waldmann P Sillanpaa MJ Bayesian analysis of genetic differentiation between populations Genetics 2003 163 367 374 12586722 10.1093/genetics/163.1.367 PMC1462429

[B39] Pella J Masuda M The Gibbs and split-merge sampler for population mixture analysis from genetic data with incomplete baselines Can J Fish Aquat Sci 2006 63 576 596 10.1139/f05-224

[B40] Huelsenbeck JP Andolfatto P Inference of population structure under a Dirichlet process model Genetics 2007 175 1787 1802 10.1534/genetics.106.061317 17237522 PMC1855109

[B41] Whittaker JC Harbord RM Boxall N Mackay I Dawson G Sibly RM Likelihood-based estimation of microsatellite mutation rates Genetics 2003 164 781 787 12807796 10.1093/genetics/164.2.781 PMC1462577

[B42] F.A.O Fisheries statistics, capture production Rome 2005 http://www.fao.org/fishery/statistics/software/fishstat/en

[B43] Frankham R Effective population size/adult population size ratios in wildlife: a review Genetic Res 1995 66 95 107 10.1017/S0016672308009695 18976539

[B44] Hauser L Adcock GJ Smith PJ Ramirez JHB Carvalho G Loss of microsatellite diversity and low effective population size in an overexploited population of New Zealand snapper (*Pagrus auratus*) P Natl Acad Sci USA 2002 99 11742 11747 10.1073/pnas.172242899 PMC129339 12185245

[B45] Hedgecock D Beaumont A Does variance in reproductive success limit effective population size of marine organisms? Genetics and evolution of aquatic organisms 1994 London, UK: Chapman and Hall 122 134

[B46] Shrimpton JM Health DD Census vs. effective population size in chinook salmon: large- and small-scale environmental perturbation effects Mol Ecol 2003 10 2571 2583 10.1046/j.1365-294X.2003.01932.x 12969462

[B47] Garza JC Wlliamson EG Detection of reduction in population size using data from microsatellite loci Molecular Ecology 2001 10 305 318 10.1046/j.1365-294x.2001.01190.x 11298947

[B48] Ryman N Palm S POWSIM: a computer program for assesing statistical power when testing for genetic differentation Mol Ecol 2006 6 600 602 10.1111/j.1471-8286.2006.01378.x

[B49] Appleyard SA Ward RD Grewe PM Genetic stock structure of bigeye tuna in the Indian Ocean using mitochondrial DNA and microsatellites J Fish Biol 2002 60 767 770 10.1111/j.1095-8649.2002.tb01701.x

[B50] Appleyard SA Grewe PM Innes BH Ward RD Population structure of yellowfin tuna (*Thunnus albacares*) in the western Pacific Ocean, inferred from microsatellite loci Mar Biol 2001 139 383 393 10.1007/s002270100578

[B51] Alvarado Bremer JR Stequert B Robertson NW Ely B Genetic evidence for inter-oceanic subdivision of bigeye tuna (*Thunnus obesus*) populations Mar Biol 1998 132 547 557 10.1007/s002270050420

[B52] Carlsson J McDowell JR Diaz-Jaimes P Carlsson JE Boles SB Gold JR Graves JE Microsatellite and mitochondrial DNA analyses of Atlantic bluefin tuna (*Thunnus thynnus thynnus*) population structure in the Mediterranean Sea Mol Ecol 2004 13 3345 3356 10.1111/j.1365-294X.2004.02336.x 15487994

[B53] Feldheim KA Gruber SH Ashley MV Population genetic structure of the lemon shark (*Negaprion brevirostris*) in the western Atlantic: DNA microsatellite variation Mol Ecol 2001 10 295 303 10.1046/j.1365-294x.2001.01182.x 11298946

[B54] Clark TB Ma L Saillant E Gold JR Microsatellite DNA markers for population-genetic studies of Atlantic bluefin tuna (*Thunnus thynnus thynnus*) and other species of genus Thunnus Mol Ecol Notes 2004 4 70 73 10.1046/j.1471-8286.2004.00572.x

[B55] Grewe PM Appleyard SA Ward RD Determining genetic stock structure of bigeye tuna in the Indian Ocean using mitochondrial DNA and DNA microsatellites Report for the Fisheries Research and Development Corporation FRDC No 97/122 Hobart: FRDC 2000

[B56] DeWoody JA Avise JC Microsatellite variation in marine, freshwater and anadromous fishes compared with other animals J Fish Biol 2000 56 461 473 10.1111/j.1095-8649.2000.tb00748.x

[B57] Waples RS Separating the wheat from the chaff: patterns of genetic differentiation in high gene flow species J Hered 1998 89 438 450 10.1093/jhered/89.5.438

[B58] Ward RD Grewe PM Carvalho GR, Pitcher TJ Appraisal of molecular genetic techniques in fisheries Molecular Genetics in Fisheries 1995 London: Chapman and Hall 29 54

[B59] Ward RD Woodwark M Skibinski DOF A comparison of genetic diversity levels in marine, freshwater and anadromous fishes J Fish Biol 1994 44 213 232 10.1111/j.1095-8649.1994.tb01200.x

[B60] Buonaccorsi VP Starkey E Graves JE Mitochondrial and nuclear DNA analysis of population subdivision among young-of-the-year Spanish mackerel (*Scomberomorus maculatus*) from the western Atlantic and Gulf of Mexico Mar Biol 2001 138 37 45 10.1007/s002270000439

[B61] Dannewitz J Maes GE Johansson L Wickstrom H Volckaert FAM Jarvi T Panmixia in the European eel: a matter of time Proc R Soc B Lond 2005 272 1129 1137 10.1098/rspb.2005.3064 PMC1559815 16024374

[B62] Gilbert-Horvath EA Larson RJ Garza JC Temporal recruitment patterns and gene flow in kelp rockfish (*Sebastes atrovirens*) Mol Ecol 2006 15 3801 3815 10.1111/j.1365-294X.2006.03033.x 17032275

[B63] Florin AB Hoglund J Absence of population structure of turbot (*Psetta maxima*) in the Baltic Sea Mol Ecol 2007 16 115 126 10.1111/j.1365-294X.2006.03120.x 17181725

[B64] Buonaccorsi VP McDowell JR Graves JE Reconciling patterns of inter-ocean molecular variance from four classes of molecular markers in blue marlin (*Makaira nigricans*) Mol Ecol 2001 10 1179 1196 10.1046/j.1365-294X.2001.01270.x 11380876

[B65] Gonzalez EG Zardoya R Isolation and characterization of polymorphic microsatellites for the sardine, *Sardina pilchardus *(Clupleidae) Mol Ecol Notes 2007 7 519 521 10.1111/j.1471-8286.2006.01640.x

[B66] Imron JB Hale P Degnan BM Degnan SM Pleistocene isolation and recent gene flow in *Haliotis asinina*, an Indo-Pacific vetigastropod with limited dispersal capacity Mol Ecol 2007 16 289 304 10.1111/j.1365-294X.2006.03141.x 17217345

[B67] Peterson RG Stramma L Upper-level circulation in the South-Atlantic Ocean Prog Oceanogr 1991 26 1 73 10.1016/0079-6611(91)90006-8

[B68] Stramma L Lutjeharms JRE The flow field of the subtropical gyre of the South Indian Ocean Journal of Geophysical Research-Oceans 1997 102 C3 5513 5530 10.1029/96JC03455

[B69] Website title "The Agulhas Current." Ocean Surface Currents http://oceancurrents.rsmas.miami.edu/atlantic/agulhas.html

[B70] Quartly GD Srokosz MA Seasonal-variations in the region of the Agulhas retroflection – studies with geosat and fram Journal of Physical Oceanography 1993 23 9 2107 2124 10.1175/1520-0485(1993)023<2107:SVITRO>2.0.CO;2

[B71] Wright S The genetical structure of populations Ann Eugen 1951 15 4 323 354 10.1111/j.1469-1809.1949.tb02451.x 24540312

[B72] O'Reilly PT Canino MF Bailey KM Bentzen P Inverse relationship between *F*_*ST *_and microsatellite polymorphism in the marine fish, walleye pollock (*Theragra chalcogramma*): implications for resolving weak population structure Mol Ecol 2004 13 1799 1814 10.1111/j.1365-294X.2004.02214.x 15189204

[B73] Bossart JL Prowell DP Genetic estimates of population structure and gene flow: limitations, lessons and new directions TREE 1998 13 202 206 21238268 10.1016/S0169-5347(97)01284-6

[B74] Gaggiotti OE Lange O Rassmann K Gliddon C A comparison of two indirect methods for estimating average levels of gene flow using microsatellite data Mol Ecol 1999 8 1513 1520 10.1046/j.1365-294x.1999.00730.x 10564457

[B75] Balloux F Goudet J Statistical properties of population differentiation estimators under stepwise mutation in a finite island model Mol Ecol 2002 11 771 783 10.1046/j.1365-294X.2002.01474.x 11972763

[B76] Balloux F Lugon-Moulin N The estimation of population differentiation with microsatellite markers Mol Ecol 2002 11 155 165 10.1046/j.0962-1083.2001.01436.x 11856418

[B77] Nei M Molecular evolutionary genetics 1987 New York: Columbia University Press

[B78] Nei M Chesser RK Estimation of Fixation Indexes and Gene Diversities Ann Hum Genet 1983 47 253 259 10.1111/j.1469-1809.1983.tb00993.x 6614868

[B79] Belkir K Borsa P Chickhi L Raufaste N Bonhomme F GENETIX 4.04 Logici el sous Windows TM, pour la Génétique des Populations 2000 Laboratoire Génome, Populations, Interactions, CNRS UMR 5000, Université de Montpellier II, Montpellier, France

[B80] Goudet J FSTAT, a Programe to Estimate and Test Gene Diversities and Fixation Indices, Version 2.9.3 2001 http://www2.unil.ch/popgen/softwares/fstat.htm

[B81] Raymond M Rousset F GENEPOP 3.3: population genetic software for exact test and ecumenism J Hered 1995 86 248 249

[B82] Guo SW Thompson EA Performing the exact test of Hardy-Weinberg proportion for multiple alleles Biometrics 1992 48 361 372 10.2307/2532296 1637966

[B83] Rice WR Analysing tables of statistical tests Evolution 1989 43 223 225 10.2307/2409177 28568501

[B84] Weir BS Cockerham CC Estimating *F*-statistics for the analysis of population structure Evolution 1984 38 1358 1370 10.2307/2408641 28563791

[B85] Slatkin M A measure of population subdivision based on microsatellite allele frequencies Genetics 1995 139 457 462 7705646 10.1093/genetics/139.1.457 PMC1206343

[B86] Excoffier L Laval G Schneider S Arlequin ver. 3.0: An integrated software package for population genetics data analysis Evol Bioinf online 2005 1 47 50 PMC2658868 19325852

[B87] Goldman SJ Rst Calc: a collection of computer programs for calculating estimates of genetic differentiation from microsatellite data and determining their significance Mol Ecol 1997 6 881 885 10.1111/j.1365-294X.1997.tb00143.x

[B88] Hardy OJ Vekemans X SPAGEDi: a versatile computer program to analyse spatial genetic structure at the individual or population levels Mol Ecol Notes 2002 2 618 620 10.1046/j.1471-8286.2002.00305.x

[B89] Excoffier L Smouse PE Quattro JM Analysis of molecular variance inferred from metric distances among DNA haplotypes: application to human mitochondrial DNA restriction data Genetics 1992 131 479 491 1644282 10.1093/genetics/131.2.479 PMC1205020

[B90] Wright S The interpretation of population-structure by *F*-statistics with special regard to systems of mating Evolution 1965 19 395 420 10.2307/2406450

[B91] Pritchard JK Stephens M Donnelly P Inference of population structure using multilocus genotype data Genetics 2000 155 945 959 10835412 10.1093/genetics/155.2.945 PMC1461096

[B92] Huelsenbeck JP Huelsenbeck ET Andolfatto P Structurama: Bayesian inference of population structure Bioinformatics 2007 in press 10.4137/EBO.S6761 PMC3118697 21698091

[B93] Beerli P MIGRATE-n a maximum likelihood program to estimate gene flow using the coalescent 2008 http://popgen.scs.fsu.edu/Migrate-n.html

